# An Up-To-Date Overview of Dental Tissue Regeneration Using Dental Origin Mesenchymal Stem Cells: Challenges and Road Ahead

**DOI:** 10.3389/fbioe.2022.855396

**Published:** 2022-04-12

**Authors:** Lin-Hong Wang, Si-Zhe Gao, Xiao-Lei Bai, Zheng-Lin Chen, Fan Yang

**Affiliations:** ^1^ Center for Plastic & Reconstructive Surgery, Department of Stomatology, Zhejiang Provincial People’s Hospital (Affiliated People’s Hospital, Hangzhou Medical College), Hangzhou, China; ^2^ Department of Stomatology, Zhejiang Chinese Medical University, Hangzhou, China; ^3^ Institute of Basic Science and Forensic Medicine, Hangzhou Medical College, Hangzhou, China; ^4^ Hangzhou Junhe Regenerative Medicine Research Center, Hangzhou, China

**Keywords:** dental, tissue regeneration, mesenchymal stem cells, proliferation rate, induced pluripotent stem cells

## Abstract

Stem cells (SCs) research has experienced exponential growth in recent years. SC-based treatments can enhance the lives of people suffering from cardiac ischemia, Alzheimer’s disease, and regenerative drug conditions, like bone or loss of teeth. Numerous kinds of progenitor/SCs have been hypothesized to depend on their potential to regain and/or heal wounded tissue and partly recover organ function. Growing data suggest that SCs (SCs) are concentrated in functions and that particular tissues have more SCs. Dental tissues, in particular, are considered a significant cause of mesenchymal stem cells (MSCs) cells appropriate for tissue regeneration uses. Tissue regeneration and SCs biology have particular attention in dentistry because they may give a novel method for creating clinical material and/or tissue redevelopment. Dental pulp, dental papilla, periodontal ligament, and dental follicle contain mesenchymal SCs. Such SCs, which must be identified and cultivated in specific tissue culture environments, may be used in tissue engineering applications such as tooth tissue, nerve regeneration, and bone redevelopment. A new cause of SCs, induced pluripotent SCs, was successfully made from human somatic cells, enabling the generation of the patient and disease-specific SCs. The dental SC’s (DSCs) multipotency, rapid proliferation rate, and accessibility make it an ideal basis of MSC for tissue redevelopment. This article discusses current advances in tooth SC investigation and its possible application in tissue redevelopment.

## Introduction

The advent of stem cells (SCs), together with current developments in molecular and cellular biology, has resulted in innovative treatment techniques aimed at regenerating several organs damaged by illness. In general, SCs possess two significant characteristics: They can renew themselves and, upon separation, generate cells with the capacity to segregate (Bianco et al., 2008; Iohara et al., 2004). Tissue engineering is a diversified science that brings together engineering, biology, and medical research to generate new organs and tissues. It is fundamentally sound science that entails the choice of suitable cells, the fabrication of scaffolds, and the use of morphogenic gestures to stimulate cells to redevelop organs or tissue (Arien-Zakay et al., 2010). Medicine has just started to study the potential uses of tissue engineering and SCs in the redevelopment of bodily components (Meirelles and Nardi, 2009). It is becoming increasingly apparent that this theoretical approach to treatment, dubbed renewing medicine, will eventually find a home in clinical practice. SCs have been demonstrated to play a critical part in coming medical therapies. They can be easily cultivated and encouraged to separate into some cell form in culture.

SCs are self-renewing cells that would develop into various specialized cells through mitosis. The embryonic SCs (ESCs) are undifferentiated cells that can grow into practically some type of cell in the body (Hemmat et al., 2010). The surrounding atmosphere is critical for sustaining a SC state. The microenvironment controls the equilibrium of self-renewal and variation. This intercellular connection between stromal cells and embryonal tumor cells has been identified, and it reflects gene expression patterns in both biological compartments (Liao et al., 2011).

Researchers can stimulate these cells to multiply in an uncontrolled manner. On the other hand, the use of ESC is contentious and involves legal and ethical issues, precluding its use in the creation of novel medicines (Hemmat et al., 2010).

One other source of SCs is the umbilical cord. The SCs in umbilical cord blood are identical to those present in a newborn infant. These cells can proliferate and develop into a series of different cell forms. After delivery, umbilical cord SCs may be Cryonic frozen for forthcoming clinical treatment (Arien-Zakay et al., 2010).

Mesenchymal SCs (MSC) are pluripotent cells, that were initially get from mature bone marrow and then form various tissues throughout grown and fetal development. Human SCs generally differentiate into the cell types found in the tissue in which they are found. Whereas research has presented that SCs from single tissue may create cells from totally other tissue (Meirelles and Nardi, 2009).

In contrast to embryonic SCs, adult SCs have the potency to be employed to cure regenerative diseases, heart ischemia, bone, and loss of teeth. SCs may be used in the future to treat tumors and Parkinson’s disease (Liao et al., 2011). Adult SCs are less contentious for research and medicinal uses since they may be obtained without harming an embryo. Neonatal SCs have been discovered in nearly every body tissue, containing dental tissues. Tissue engineering capacity has been recognized in dental SCs (Demarco et al., 2011). They provide the possibility for use in SC therapy because of their pluripotent differentiation capacity, thus they may be used to promote the regeneration of non-dental tissues, for example, nerves and bones (Nör, 2006; Demarco et al., 2011).

The induced pluripotent stem cells (iPS) cells are a new SC derived from human somatic cells (Takahashi et al., 2007a; Warren et al., 2010). iPS cells are similar to human ESC and can develop into sophisticated derivatives of all three major germ layers. Unlike ESC, iPS cells can be used to create patient-specific SCs, which can generate tissue-matched differentiating donor cells for fundamental investigation, ailment models, and redeveloping drugs (Warren et al., 2010). This innovation may usher in a new age of individualized medicine.

This study examines the current state of the area of regenerative medicine based on SCs, focusing on the bases of SCs found in dental tissues and recent discoveries in the area of dental SC (DSC) study and its prospective application in tooth bioengineering.

Several different SC populations have been extracted from various parts of the teeth. Adult SCs have been discovered in the tooth pulp since 2000 (Gronthos et al., 2000), several other forms of DSC, such as those produced from highly porous tooth eruption, have been identified from adult and immature teeth (Miura et al., 2003), SCs produced from the papilla apicalis (Sonoyama et al., 2008), MSC derived from tooth germs (Morsczeck et al., 2009) and SCs derived from human PDL (periodontal ligament) (Seo et al., 2004). These SCs are assumed to be immature mesenchymal cells present in oral tissues with an unlimited potential to self-renew, colony development, and develop into several forms (Bianco et al., 2008). Several characteristics of these recently found DSCs have been likened to those of bone marrow-derived stromal SCs (BMDSCs). DSC has the ability for multi-differentiation, giving birth to diverse cell lineages such as adipogenic, neurogenic, and osteo/osteogenic. As a result, these cells have been subjected to bioengineering investigations to determine their use in preclinical studies (Demarco et al., 2011).

It is critical to remember, that various kinds of dental-tissue-derived MSC share some traits; they also exhibit substantial variability, as seen by significant phenotypic distinctions that most likely represent diverse functional qualities (Bianco et al., 2008). There is already evidence of substantial diversity in the odontogenic strength of single colony-derived populations separated from the tooth pulp, owing to heterogeneity in the patterns of its protein expression and genotypic (Waddington et al., 2009). Additionally, this variability can be exacerbated greatly due to their tissue microenvironment (Vêncio et al., 2011). This problem gets more difficult since investigators have isolated and cultured dental MSC in various ways and evaluated their differentiation capacity.

### Dental Pulp SCs

DPSCs were the initial SCs obtained from mature human dental pulp. They were obtained from permanent third molars and displayed a high rate of colony formation and propagation, which resulted in the creation of calcified nodules (Gronthos et al., 2000). *In vitro*, DPSC cultures from affected third molars at the root development stage differentiated into odontoblast-like cells with a high propensity for migration and mineralization, resulting in structured three-dimensional (3D) dentin-like structures (Bakopoulou et al., 2011).

**FIGURE 1:**
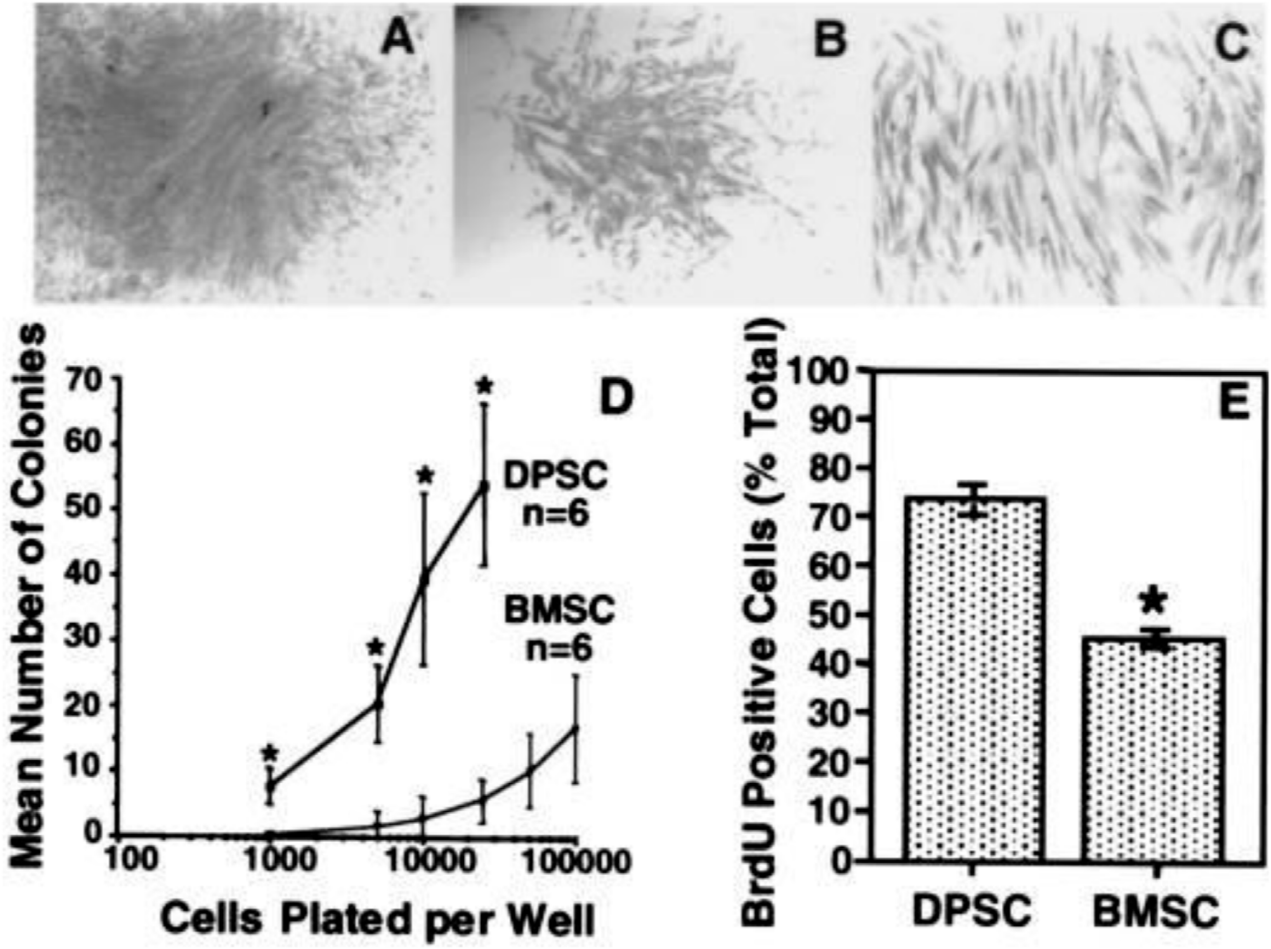
*In vitro* colony formation efficiency and cell proliferation. After 14 days, representative colonies with a high **(A)** and a low **(B)** density. The appearance is characteristic of fibroblast-like cells **(C)**. The frequency of colony-forming cells in the dental pulp tissue and bone marrow at numerous plating densities suggests that dental pulp has a greater number of clonogenic cells than bone marrow **(D)**. BrdUrd-positive cells were represented as a proportion of DPSCs and BMSCs enumerated (Gronthos et al., 2000).

Colonies in DPSC exhibit varying cell densities, implying that every cell clone can develop at a different pace (Gronthos et al., 2000) in the same population; cells with various shapes and sizes can be detected (Figure 1). The variation of DPSC into a particular cell lineage is primarily influenced by microenvironmental factors for example receptor molecules, growth factors, signaling molecules, extracellular matrix proteins, and transcription factors. Odontoblast, myocyte, chondrocyte, neurocyte, adipocyte, melanoma cell, osteoblast, iPS, corneal epithelial cell are among the cell types that may be regenerated from DPSC (Stevens et al., 2008; Yan et al., 2010). Almushayt et al. (Almushayt et al., 2006) revealed a non-collagen extracellular matrix protein excerpt from dentin. DMP1 may greatly enhance DPSC differentiation into odontoblasts and the development of tissue repair on uncovered pulp tissue. Additionally, when cured with transforming growth factor 1 (TGF1) alone or in conjunction through FGF2, DPSC may be induced into the odontoblast lineage (He et al., 2008).

Dentin is histologically distinct from tooth pulp, although they are inextricably linked. DPSC can repair dentin and supply oxygen, nourishment, and stimulation, while strong dentin protects fragile dental pulp tissue. They work in tandem to preserve the form and function of teeth. Any physiological or pathological response, for instance, caries, trauma, or cavity preparation, will affect the other. Both of these form a dentin-pulp complex and contribute to the tooth’s different biological functions. Numerous studies have shown that DPSC is critical for dentin-pulp tissue repair (Gronthos et al., 2000). DPSC revealed the capacity to create functional tooth tissue in the shape of pulp-like/dentin aggregates after *in vivo* transplantation (Gronthos et al., 2002). In immunocompromised mice, *ex vivo* transplanted DPSC combined with tricalcium phosphate/hydroxyapatite create ectopic pulp-like/dentin aggregates. These clusters of heterogeneous DPSC generate vascularized pulp-like tissue bounded by a sheet of odontoblast-like cells that express factors that result in the formation of dentin-containing tubules comparable to those observed in genuine dentin (Gronthos et al., 2002; Batouli et al., 2003). A previous study (Huang et al., 2010) showed that DPSC may regenerate a dentin-pulp-like complex with well-known vascularity in empty root canal space. These investigations signify important advancement in the future preservation of pulp tissue and developing a new biological therapy option for endodontic disorders.

Additionally, DPSC may produce neural biomarkers and develop into operationally nerve cells, indicating their effectiveness in neuronal illnesses as a cellular treatment (Nör, 2006). DPSC was implanted into cerebrospianal fluid (CSF) of rats with cortical damage in recent research. These cells moved as single cells into several brain areas and were discovered expressing neuron-specific markers in the damaged cortex. This demonstrated that DPSC-derived cells might assimilate into the host brain and act as a source of gliogenesis and neuro *in vivo*, particularly when the brain is damaged (Király et al., 2011). The ability of these cells to differentiate spontaneously extremely implies that they might be used in restructuring medicine.

### Human Separated Deciduous Teeth SCs

**FIGURE 2:**
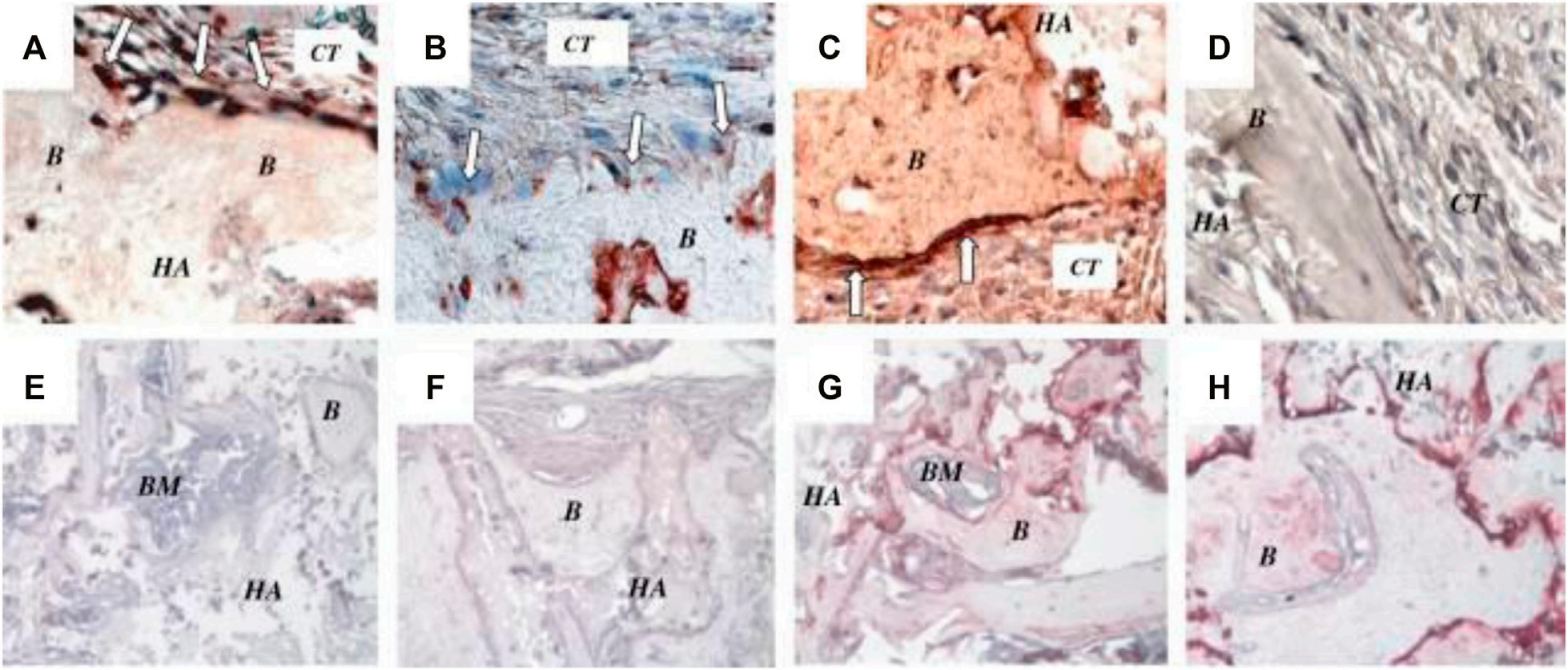
*In vivo* characterization of SHED. **(A)** After 8 weeks transplantation in immunocompromised mice, SHED were able to form bone **(B)** on the surface of hydroxyapatite/tricalcium phosphate (HA). Osteogenic cells were positive for anti-ALP (open arrows in **A**), BSP (open arrows in **B**) and type I collagen (open arrows) in **(C)** antibody staining revealed osteogenic cells. BSP and type I collagen stain positively on connective tissue (CT) cells. **(D)** Immunohistochemical staining of SHED transplants with preimmune serum as a negative control. DSP was not expressed as an odontogenic marker in bone marrow mesenchymal SCs **(E)** or SHED **(F)**. BSP expression was positive in bone marrow mesenchymal SC-mediated bone **(G)** as well as bone marrow SHED-mediated bone (g) **(H)**. BM stands for bone marrow, HA for HA/TCP. Reproduced with permission from (Seo et al., 2008).

Additionally, SCs can be extracted from the pulp of exfoliated deciduous teeth in humans (SHED). *In vitro*, these cells may induce bone growth, synthesize dentin, and develop into non-dental mesenchymal cell variants. SHED demonstrates accelerated propagation rates, colony doublings, osteoinductive capabilities *in vivo*, and the capacity to cluster in sphere-like clusters. They, still, are unable, unlike DPSCs, to redevelop full pulp-like/enamel aggregates *in vivo* (Gronthos et al., 2000). SHED’s osteoinductive properties enable it to heal serious calvarial abnormalities in mice with significant bone production (Seo et al., 2008) (Figure 2). Due to their potential to create and release neurotrophic factors, dental SCs may also be useful in treating neurodegenerative diseases and in the regeneration of motor neurons after injury, showing that dental SCs generated from primary molars produce neural markers for instance nestin (Govindasamy et al., 2010). The production of a neural factor in dental SCs piques interest in their possible use in brain regeneration, such as Parkinson’s disease therapy. Researchers continue to investigate dental SCs’ effectiveness in non-dental redevelopment.

### Apical Papilla SCs

Recently, the morphological dental papilla histological properties found at the apex of growing human permanent teeth were reported; this tissue was dubbed apical papilla. This tissue is only tenuously linked to the growing root’s tip and is readily removed. At the top of the tooth root, a colony of SCs separated from human teeth was discovered. These cells, dubbed SCs from the apical papilla (SCAP), have developed and proliferated at a higher rate *in vitro* than DPSC. Between the pulp and the apical papilla lies an apical cell-rich zone. Notably, progenitor/SCs were found in the apical papilla and the dental pulp, although their features are considerably different (Sonoyama et al., 2008). Due to SCAP’s increased proliferative capacity, this colony of cells is well suited for cell-based redevelopment and, more specifically, for root formation. They are skillful at generating enamel and odontoblast-like cells production *in vivo* and are thus possible to be the cause of most important odontoblasts for root enamel creation (Sonoyama et al., 2008). SCAP can also elucidate a scientific phenomenon defined in various latest medical state reports: apexogenesis may develop in diseased undeveloped permanent teeth suffering from apical periodontitis (Chueh and Huang, 2006). Given the closeness to the periapical tissues, SCAP living in the apical papilla probably endured the contagion. This tissue may benefit from its secondary circulation, which allows it to continue the pulp necrosis process. Maybe, after endodontic cleaning, these cells differentiate into the most important odontoblasts that finish the root development process.

### SCs of the Periodontal Ligament

**FIGURE 3:**
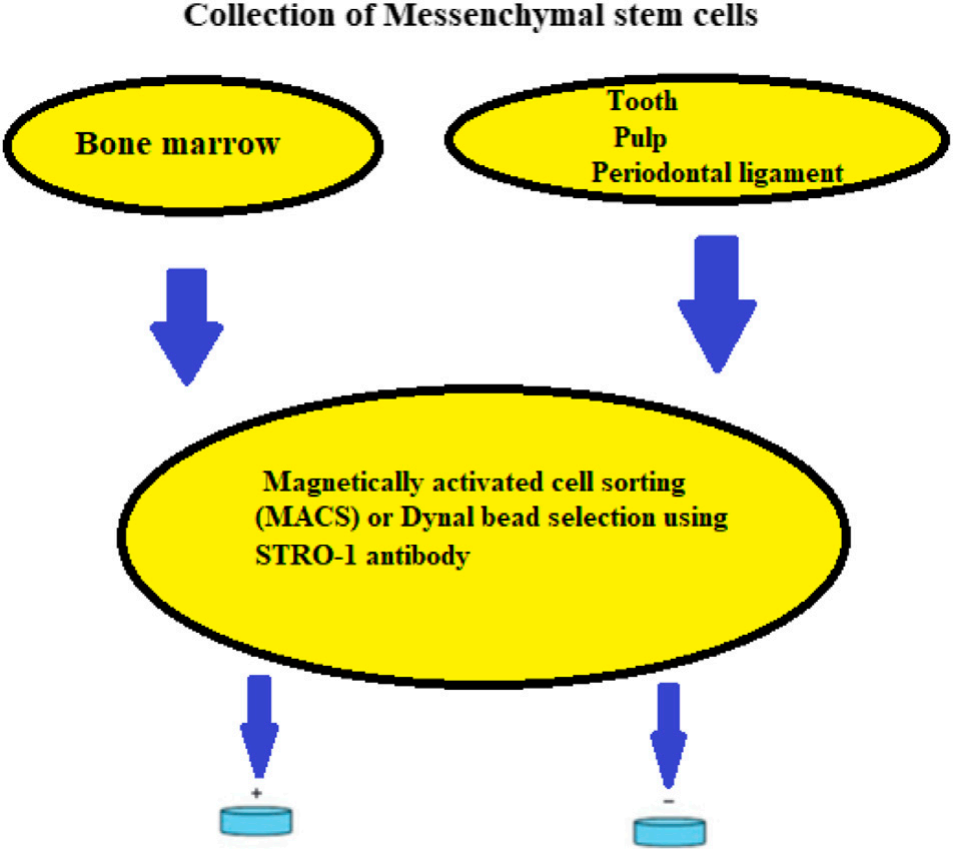
Isolation of mesenchymal SCs from bone marrow, dental pulp, and periodontal ligament using a schematic representation.

**FIGURE 4:**
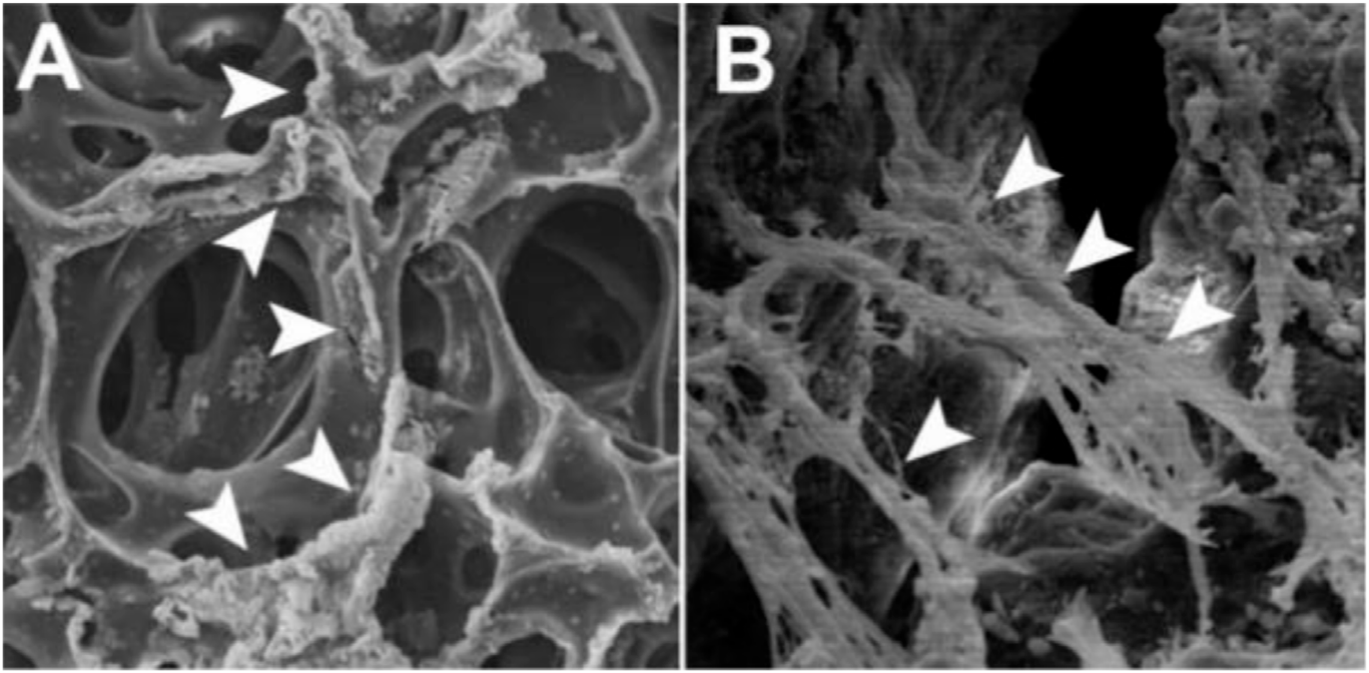
On top of the polymers, SEM micrographs of human PDL-MSCs **(A)** Numerous crushed cell colonies seem to be linked mostly by fibrin sponge surface hollows (arrowheads) **(B)** Tight connections between cells and substrate are established by the growth of cytoplasmic processes and filopodia that serve as anchors for the cells. At their distal extremities, flopodia and cytoplasmic processes are closely connected to biomimetic molecules (arrowheads). Reproduced with permission from (Trubiani et al., 2008).

The periodontal ligament (PDL) is a gap among the alveolar and cementum that substitutes the follicular area that surrounds the growing tooth throughout the cap and bud phases of development. Sharpey’s fibers or cementoblast fibers may be injected into the cementum layer (in cellular intrinsic fiber cementum). During tooth eruption, the PDL grows, ready to sustain the functioning tooth against occlusal stresses. Major collagen bundles cover the whole mature PDL and are embedded in both alveolar bone and cementum. Fibers are oriented in precise ways to enhance the absorption of pressures applied to the tooth throughout mastication. The PDL has been known as a source of neural SCs, and new research has discovered a colony of SCs capable of developing via mesenchymal cell pathways to generate connective tissue rich in collagen adipocytes and cementoblast-like cells (Figure 3) (Seo et al., 2004). Related to bone marrow stromal SCs and DPSC, PDL SCs exhibit cell surface marker features and variation capacity (Seo et al., 2004). PDL-like/cementum structures were generated after PDLSC transplantation into immunocompromised animals. *Ex vivo* expanded human PDLSC sown in 3D scaffolds (bovine-derived replacements and fibrin sponge) generated bone (Trubiani et al., 2008) (Figure 4). Additionally, these cells have been demonstrated to maintain SC characteristics and the ability for tissue redevelopment. These results imply that this colony of cells may be employed to build a biological root comparable to a metal graft by covering it with a synthetic dental crown.

### The Dental Follicle’s Precursor Cells

The dental follicle is a thin band of connective tissue that covers the growing tooth. The dental follicle has long been regarded as a pluripotent tissue because of its potential to form bone, collagen fibers, and PDL from fibrous tissue generated from ectomesenchyme. DFPC may be identified and cultured under controlled tissue culture situations, and their present characterization has raised their strength for use in bioengineering applications such as bone redevelopment and periodontal (Lin et al., 2000; Sonoyama et al., 2008). DFPC differentiate into PDL fibroblasts that release collagen and contact surrounding cementum fibers and bone. Dental follicle neural SCs gained from human third molars are distinguished by their capacity to form compressed calcified nodules *in vitro* and their fast adhesion in culture (Lin et al., 2000). DFPC, like SCAP, signifies cells from a growing tissue and may therefore have a stronger potential for differentiation than former DSCs. On the other hand, like with SCAP, more study on the features and possible applications of these cells is required (Table 1).

**TABLE 1:** Stem cell type in the dental cells.

Properties	Stem cells from the apical paplilla	Periodontal ligament stem cells	Dental pulp stem cells	Stem cells from the pulp of human exfoliated deciduous teeth	Dental follicle precursor cells
Location	Apical papilla of emerging root	Periodontal ligament	Permanent tooth pulp	Exfoliated deciduous tooth pulp	Dental follicle of emerging tooth
Propagation rate	High	High	Moderate	High	High
	—	Yes	—	—	—
Multipotentiality	Odontoblast, adipocyte, neurocyte, iPS osteoblast	Odontoblast, chondrocyte	Odontoblast, myocyte, chondrocyte	Osteoblast, odontoblast	Odontoblast, neurocyte osteoblast
		osteoblast	neurocyte	myocyte, Induced pluripotent stem cell	
		cementoblast, neurocyte	adipocyte	neurocyte, chondrocyte	
			melanoma cell, osteoblast	adipocyte	
			Induced pluripotent stem cell, corneal		
			epithelial cell		
Tissue repair	Bone redevelopment, dentin-pulp restoration, root development	Bone redevelopment, periodontal regeneration root development	Neuroregeneration, bone redevelopment	Bone redevelopment, tubular dentin neuroregeneration	Periodontal regeneration, bone
	neuroregeneration		myogenic		regeneration
			redevelopment, dentin pulp restoration		
Heterogeneity	Yes	Yes	Yes	Yes	Yes

### Dental Tissue Engineering and Dental Pulp SCs

In a variety of research disciplines, DSC is presently regarded as having the capacity for tissue repair. These contain the evident function of cells to heal PDL, dental pulp, and dentin (Huang et al., 2010; Demarco et al., 2011). Even DSCs are being used as a source of cells to aid in the redevelopment of extra tissues for instance bone and nerves are being investigated (Nör, 2006; Seo et al., 2008; Demarco et al., 2011). Attempts to stimulate tissue repair in the pulp space have been ongoing for some time. Ostby (Nygaard-östby and Hjortdal, 1971) advocated causing bleeding and the production of blood clots in the canal space of adult teeth in 1962 to direct tissue healing in the canal. On the other hand, the connective tissue that expanded into the stream area was restricted in size, and its origin is unknown. Restorative Endodontics is a novel cure method that relies on restoring functional status and root growth. This therapeutic method is based on the endodontic administration of a thrombus (scaffold), growth factors (perhaps derived from dentin and platelets), and progenitors (Lovelace et al., 2010). The latest research established that MSC is transported into root canal regions throughout renewing endodontic operations on young teeth with open apices (Lovelace et al., 2010). These results offer a physiologic foundation for SCs to participate in the continuing root growth and restorative response after this therapeutic operation.

Due to DPSC’s powerful dentinogenic capacity, they may be employed in critical pulp treatment. When DPSCs are implanted single or in conjunction with BMP2 into the pulp cavity, they may considerably enhance dentin-pulp-like complex healing and rebuilding (Nygaard-östby and Hjortdal, 1971; Iohara et al., 2004). Another study (Prescott et al., 2008) implanted the trio of DPSC, DMP1, and a collagen scaffold, into dentin slices to represent perforation sites and then subcutaneously implanted the replication into naked mice. Following a 6-week incubation period, it was possible to identify well-organized pulp-like tissue at the puncture site. A previous study (Cordeiro et al., 2008) showed that SHED/scaffold remixing in the human tooth segment might also result in the formation of dental pulp-like configurations. A previous study (Huang et al., 2010) shows that either DPSC or SHED may regenerate enamel-pulp-like complicated with well-known vascularity in empty root canal space (Huang et al., 2010). The trickiest part of designing a regenerative endodontic treatment is how the different processes may be improved and combined to create a restored pulp-dentin complex. The growth of redevelopment endodontic techniques will need an extensive study program examining these elements and their therapeutic use.

Adult periodontitis has been the best frequent cause of loss of teeth since it results from permanent loss of alveolar bone stability and connective tissue attachment. The difficulty for cell-based restoration of a functioning periodontium and so to produce new bone and ligament and to guarantee that the proper influences among these tissues, as well as among the tooth root and bone, are established. This is not a simple task since they are highly distinct tissues that grow in an orderly (spatial and temporal) way throughout tooth formation (Wolf and Lamster, 2011). In latest years, targeted tissue repair has established itself as the gold standard procedure for regenerating periodontist tissue. This surgery stretches a biocompatible membrane from the root surface to the neighboring alveolar bone over the periodontal deficiency, sometimes in conjunction with a bone transplant (AlGhamdi et al., 2010). The border membrane keeps undesirable periodontal and epithelium connective tissue out of the repair site, allowing PDL cells to repopulate the damaged region. Due to the minimal relative effectiveness of this procedure, researchers have developed strategies to increase it, such as the inclusion of exogenous growth hormones and SC treatment (Chen et al., 2008). One objective of the present investigation is to use a distinct colony of dental SCs to mimic important processes in periodontal growth both geographically and temporally to facilitate reengineering of the periodontium and its sequential repair (Sonoyama et al., 2006).

Growth factors such as emdogain, platelet-derived growth factor, bone morphogenetic proteins, and recombinant amelogenin protein are often employed in PDL regeneration therapy. The enhanced regeneration capacity that results might be a consequence of greater recruitment of progenitor MSC that differentiate into PDL tissue. PDLSC treated with bone morphogenetic protein expression vectors and platelet-derived growth factors were recently studied in periodontal bioengineering models (Zaman et al., 1999). These investigations demonstrated that normal periodontal tissues, including alveolar bone, organized cementum, and the PDL attachment machinery, may regenerate. Additionally, the feasibility of building a root-periodontal tissue complex was established effectively by implanting and growing a pelleted tricalcium phosphate/hydroxyapatite scaffold including SCAP covered with PDLSC-seeded Gelfoam in a minipig tooth cavity (Miura et al., 2003; He et al., 2010). By building multilayered cell sheets supported by woven polyglycolic acid, the multipotent differentiation features of PDLSC for the generation of both soft and hard tissues were demonstrated more. When transplanted cells seeded polyglycolic acid sheets were placed into root surfaces, they restored new cementum, bone, and well-oriented collagen fibers. Along with PDL-derived DSCs, a previous study reported that MSC and SCs generated from adipose tissue enhance periodontal tissue repair (Taba et al., 2005).

In a present work (Park et al., 2011), three types of adult SCs generated from dental tissue were isolated from excised juvenile canine molars, and *ex vivo* grown PDLSC, DPSC, and periapical follicular SCs were implanted into the apical participation deficit. Allogeneic PDLSC had the greatest ability to regenerate PDL, cementum, and alveolar bone, along with blood vessel and peripheral nerve, as determined by conventional and immunological histology.

Efficient PDL tissue repair therapies will enhance periodontal disease therapy and can also be utilized to increase present dental implant treatments. Several efforts to regenerate the extracellular matrix surrounding dental transplants have demonstrated the difficulty of preventing fibrous tissue encapsulation and creating usable cambium on the implant’s surface (Lin et al., 2009).

### Dental MSCs-Based Clinical Trials

Numerous research deploying MSCs to repair orofacial bones have previously been reported, such as sinus augmentation and regeneration of large- or small-size bone abnormalities (alveolar ridge augmentation, cleft palate, mandibular fracture, replacement, osteoradionecrosis cases, and maxillary replacement). Previous studies (Jakobsen et al., 2013; Padial-Molina et al., 2015) performed systematic evaluations of these research, the majority of that were case reports/series with a few randomized controlled clinical trials (RCTs). The majority of this study used Bone marrow stromal cells (BMSCs) and, to a lesser degree, other MSC types such as periosteum-derived MSCs or adipose tissue-derived MSCs. Before cell transplantation, these cells were plated in a growth medium containing autologous serum, bovine serum, or other growth media and were either preinduced to differentiate into osteogenic cells.

By contrast, just a few clinical studies with oral MSCs have been reported yet. In two consecutive studies, Papaccio et al. focused on autologous DPSCs in conjunction with a collagen sponge to heal human mandible bone injuries after third molar extraction (d'Aquino et al., 2009; Giuliani et al., 2013; Papaccio et al., 2006). Three months after surgery, the authors discovered complete vertical bone healing and periodontal tissue regeneration to the second molars. Additionally, they explore bone quality 3 years after transplantation and observed that the outcome was entirely compact rather than porous bone, with no significant clinical implications. Significantly, all trials were undertaken in the absence of the previously indicated internationally recognized requirements for GMP-compliant DPSC manufacturing. Nakashima et al. (Nakashima and Iohara, 2014) reported on a series of tests testing the capability of mobilized DPSCs to regenerate pulp in dog pulpectomized teeth, and based on these results, they started clinical research that is now pending approval. This will give information on the clinical feasibility of dental MSC-based pulp regeneration.

### Future Prospects

Before the improvement of successful cellular-based therapeutics for recovering drugs, many major goals must be met: Understanding self-renewal processes can let us control adult SC proliferation *in vitro* to create adequate cell numbers for various purposes. One possibility is to use nuclear transfer technology to generate ESCs. This procedure, however, utilizes unfertilized donor eggs and rejected embryos. One more strategy is to manipulate SCs *in vitro* to maintain their state. The latest examples of reprogramming somatic cells to resemble embryonic SCs with just three to four variables shed insight on the possibilities of turning cells into PSCs for a range of uses (Takahashi et al., 2007b; Yu et al., 2007; Nakagawa et al., 2008). Recognize the control of SCs throughout differentiation and establishing distinct tissues. Certain tissues, for example, cartilage, dentin, bone, and tendon, need the creation of particular extracellular materials. The Cascades of signals are activated sequentially throughout the extracellular matrix formation and its development into specialized tissues. Controlling and artificially supplying these indicators at a certain phase may enhance the intended tissue restoration (Kolf et al., 2007). Recognize how SCs and the immune system interact. Immunosuppressive allogeneic MSCs can be a readily available source of cells for therapeutic purposes. However, immunological responses should be considered, as displayed by many *in vivo* investigations (Poncelet et al., 2007). Additional study is required to evaluate if allogenic dental MSCs may reduce recipient host immunorejection in the long and short term. Governing and inhibiting the transformation of *ex vivo* expanded MSCs. It is critical to closely monitor and observe this potential since data suggests that adipose-derived MSCs have lost their genetic stability throughout time and are susceptible to tumor development (Rubio et al., 2005).

## Conclusion

There is still much to understand about dental stem/progenitor cells’ nature, potential, and activity. On the other hand, the potential for their use in dental tissue redevelopment is enormous and will result in significant gains for the treatment of dental disease’s impacts. Dental SCs exhibit various characteristics, such as a high proliferation rate, the capacity to differentiate into many cell lineages, ease of access, high viability, and can be driven to differentiate into diverse cell lineages.

As a result, these cells have been employed in ample animal tissue engineering research to examine their potential for preclinical applications. Although considerable advances have been achieved in SC research so far, their effectiveness and usefulness in clinical trials remain unknown. Before scientists get into clinical trials, they must thoroughly study the underlying knowledge and biology of SCs. MSC and iPS cell technologies may usher in a new age of individualized treatment. The variability of patient characteristics and the biology of various SC types emphasizes the use of an individualized strategy for SC treatment and other cell-based therapies.

## References

[B1] AlGhamdi A. S. Shibly O. Ciancio S. G. (2010). Osseous Grafting Part I: Autografts and Allografts for Periodontal Regeneration-Aa Literature Review. J. Int. Acad. Periodontol. 12, 34–38. 20465029

[B2] Almushayt A. Narayanan K. Zaki A. E. George A. (2006). Dentin Matrix Protein 1 Induces Cytodifferentiation of Dental Pulp Stem Cells into Odontoblasts. Gene Ther. 13, 611–620. 10.1038/sj.gt.3302687 16319946

[B3] Arien-Zakay H. Lazarovici P. Nagler A. (2010). Tissue Regeneration Potential in Human Umbilical Cord Blood. Best Pract. Res. Clin. Haematol. 23, 291–303. 10.1016/j.beha.2010.04.001 20837341

[B4] Bakopoulou A. Leyhausen G. Volk J. Tsiftsoglou A. Garefis P. Koidis P. (2011). Comparative Analysis of *In Vitro* Osteo/odontogenic Differentiation Potential of Human Dental Pulp Stem Cells (DPSCs) and Stem Cells from the Apical Papilla (SCAP). Arch. Oral Biol. 56, 709–721. 10.1016/j.archoralbio.2010.12.008 21227403

[B5] Batouli S. Miura M. Brahim J. Tsutsui T. W. Fisher L. W. Gronthos S. (2003). Comparison of Stem-Cell-Mediated Osteogenesis and Dentinogenesis. J. Dent. Res. 82, 976–981. 10.1177/154405910308201208 14630898

[B6] Bianco P. Robey P. G. Simmons P. J. (2008). Mesenchymal Stem Cells: Revisiting History, Concepts, and Assays. Cell Stem Cell 2, 313–319. 10.1016/j.stem.2008.03.002 18397751 PMC2613570

[B7] Chen Y.-L. Chen P. K.-T. Jeng L.-B. Huang C.-S. Yang L.-C. Chung H.-Y. (2008). Periodontal Regeneration Using *Ex Vivo* Autologous Stem Cells Engineered to Express the BMP-2 Gene: an Alternative to Alveolaplasty. Gene Ther. 15, 1469–1477. 10.1038/gt.2008.131 18701911

[B8] Chueh L. Huang G. (2006). Immature Teeth with Periradicular Periodontitis or Abscess Undergoing Apexogenesis: a Paradigm Shift. J. Endodontics 32, 1205–1213. 10.1016/j.joen.2006.07.010 17174685

[B9] Cordeiro M. M. Dong Z. Kaneko T. Zhang Z. Miyazawa M. Shi S. (2008). Dental Pulp Tissue Engineering with Stem Cells from Exfoliated Deciduous Teeth. J. Endodontics 34, 962–969. 10.1016/j.joen.2008.04.009 18634928

[B10] d'Aquino R. De Rosa A. Lanza V. Tirino V. Laino L. Graziano A. (2009). Human Mandible Bone Defect Repair by the Grafting of Dental Pulp Stem/progenitor Cells and Collagen Sponge Biocomplexes. Eur. Cel. Mater. 18, 75–83. 10.22203/ecm.v018a07 19908196

[B11] Demarco F. F. Conde M. C. M. Cavalcanti B. N. Casagrande L. Sakai V. T. Nör J. E. (2011). Dental Pulp Tissue Engineering. Braz. Dent. J. 22, 3–13. 10.1590/s0103-64402011000100001 21519641 PMC3736569

[B12] Giuliani A. Manescu A. Langer M. Rustichelli F. Desiderio V. Paino F. (2013). Three Years after Transplants in Human Mandibles, Histological and In-Line Holotomography Revealed that Stem Cells Regenerated a Compact rather Than a Spongy Bone: Biological and Clinical Implications. Stem Cell Translational Med. 2 (4), 316–324. 10.5966/sctm.2012-0136 PMC3659838 23502599

[B13] Govindasamy V. Abdullah A. N. Sainik Ronald V. Musa S. Che Ab. Aziz Z. A. Zain R. B. (2010). Inherent Differential Propensity of Dental Pulp Stem Cells Derived from Human Deciduous and Permanent Teeth. J. Endodontics 36, 1504–1515. 10.1016/j.joen.2010.05.006 20728718

[B14] Gronthos S. Brahim J. Li W. Fisher L. W. Cherman N. Boyde A. (2002). Stem Cell Properties of Human Dental Pulp Stem Cells. J. Dent. Res. 81, 531–535. 10.1177/154405910208100806 12147742

[B15] Gronthos S. Mankani M. Brahim J. Robey P. G. Shi S. (2000). Postnatal Human Dental Pulp Stem Cells (DPSCs) *In Vitro* and Invivo. Proc. Natl. Acad. Sci. 97, 13625–13630. 10.1073/pnas.240309797 11087820 PMC17626

[B16] He H. Yu J. Cao J. E L. Wang D. Zhang H. (2010). Biocompatibility and Osteogenic Capacity of Periodontal Ligament Stem Cells on nHAC/PLA and HA/TCP Scaffolds. J. Biomater. Sci. Polym. Ed. 22, 179. 10.1016/j.neuint.2011.01.006 20557694

[B17] He H. Yu J. Liu Y. Lu S. Liu H. Shi J. (2008). Effects of FGF2 and TGFβ1 on the Differentiation of Human Dental Pulp Stem Cells *In Vitro* . Cel. Biol. Int. 32, 827–834. 10.1016/j.cellbi.2008.03.013 18442933

[B18] Hemmat S. Lieberman D. Most S. (2010). An Introduction to Stem Cell Biology. Facial Plast. Surg. 26, 343–349. 10.1055/s-0030-1265015 20853224

[B19] Huang G. T.-J. Yamaza T. Shea L. D. Djouad F. Kuhn N. Z. Tuan R. S. (2010). Stem/progenitor Cell-Mediated De Novo Regeneration of Dental Pulp with Newly Deposited Continuous Layer of Dentin in an *In Vivo* Model. Tissue Eng. A 16, 605–615. 10.1089/ten.tea.2009.0518 PMC2813150 19737072

[B20] Iohara K. Nakashima M. Ito M. Ishikawa M. Nakasima A. Akamine A. (2004). Dentin Regeneration by Dental Pulp Stem Cell Therapy with Recombinant Human Bone Morphogenetic Protein 2. J. Dent. Res. 83, 590–595. 10.1177/154405910408300802 15271965

[B21] Jakobsen C. Sørensen J. A. Kassem M. Thygesen T. H. (2013). Mesenchymal Stem Cells in Oral Reconstructive Surgery: a Systematic Review of the Literature. J. Oral Rehabil. 40 (9), 693–706. 10.1111/joor.12079 23834336

[B22] Király M. Kádár K. Horváthy D. B. Nardai P. Rácz G. Z. Lacza Z. (2011). Integration of Neuronally Predifferentiated Human Dental Pulp Stem Cells into Rat Brain *In Vivo* . Neurochem. Int. 59, 371–381. 10.1016/j.neuint.2011.01.006 21219952

[B23] Kolf C. M. Cho E. Tuan R. S. (2007). Mesenchymal Stromal Cells. Biology of Adult Mesenchymal Stem Cells: Regulation of Niche, Self-Renewal and Differentiation. Arthritis Res. Ther. 9, 204. 10.1186/ar2116 17316462 PMC1860068

[B24] Liao Y. Geyer M. B. Yang A. J. Cairo M. S. (2011). Cord Blood Transplantation and Stem Cell Regenerative Potential. Exp. Hematol. 39, 393–412. 10.1016/j.exphem.2011.01.002 21238533

[B25] Lin C. Dong Q.-S. Wang L. Zhang J.-R. Wu L.-A. Liu B.-L. (2009). Dental Implants with the Periodontium: a New Approach for the Restoration of Missing Teeth. Med. Hypotheses 72, 58–61. 10.1016/j.mehy.2008.08.018 18829177

[B26] Lin N. H. Gronthos S. Bartold P. M. (2000). Stem Cells and Future Periodontal Regeneration. Periodontol. 51, 239–251. 10.1111/j.1600-0757.2009.00303.x 19878478

[B27] Lovelace T. W. Henry M. A. Hargreaves K. M. Diogenes A. (2010). Evaluation of the Delivery of Mesenchymal Stem Cells into the Root Canal Space of Necrotic Immature Teeth after Clinical Regenerative Endodontic Procedure. J. Endod. 37, 133–138. 10.1016/j.joen.2010.10.009 21238791

[B28] Meirelles L. d. S. Nardi N. B. (2009). Methodology, Biology and Clinical Applications of Mesenchymal Stem Cells. Front. Biosci. 14, 4281–4298. 10.2741/3528 19273350

[B29] Miura M. Gronthos S. Zhao M. Lu B. Fisher L. W. Robey P. G. (2003). SHED: Stem Cells from Human Exfoliated Deciduous Teeth. Proc. Natl. Acad. Sci. 100, 5807–5812. 10.1073/pnas.0937635100 12716973 PMC156282

[B30] Morsczeck C. Petersen J. Völlner F. Driemel O. Reichert T. Beck H. C. (2009). Proteomic Analysis of Osteogenic Differentiation of Dental Follicle Precursor Cells. Electrophoresis 30, 1175–1184. 10.1002/elps.200800796 19288589

[B31] Nakagawa M. Koyanagi M. Tanabe K. Takahashi K. Ichisaka T. Aoi T. (2008). Generation of Induced Pluripotent Stem Cells without Myc from Mouse and Human Fibroblasts. Nat. Biotechnol. 26, 101–106. 10.1038/nbt1374 18059259

[B32] Nakashima M. Iohara K. (2014). Mobilized Dental Pulp Stem Cells for Pulp Regeneration: Initiation of Clinical Trial. J. Endodontics 40 (4), S26–S32. 10.1016/j.joen.2014.01.020 24698690

[B33] Nör J. E. (2006). Buonocore Memorial Lecture. Oper. Dent 31, 633–642. 10.2341/06-000 22077641

[B34] Nygaard-östby B. Hjortdal O. (1971). Tissue Formation in the Root Canal Following Pulp Removal. Eur. J. Oral Sci. 79, 333–349. 10.1111/j.1600-0722.1971.tb02019.x 5315973

[B35] Padial-Molina M. O'Valle F. Lanis A. Mesa F. Dohan Ehrenfest D. M. Wang H. L. (2015). Clinical Application of Mesenchymal Stem Cells and Novel Supportive Therapies for Oral Bone Regeneration. Biomed. Res. Int. 2015, 341327. 10.1155/2015/341327.341327 26064899 PMC4443638

[B56] Papaccio G. Laino G. (2006). First International Meeting on “Stem Cell Applications in the Craniofacial Region”. J. Cell Physiol. 208 (3), 473–5. 10.1002/jcp.20690 16715487

[B36] Park J.-Y. Jeon S. H. Choung P.-H. (2011). Efficacy of Periodontal Stem Cell Transplantation in the Treatment of Advanced Periodontitis. Cel. Transpl. 20, 271–286. 10.3727/096368910X519292 20719084

[B37] Poncelet A. J. Vercruysse J. Saliez A. Gianello P. (2007). Although Pig Allogeneic Mesenchymal Stem Cells Are Not Immunogenic *In Vitro*, Intracardiac Injection Elicits an Immune Response *In Vivo* . Transplantation 83, 783–790. 10.1097/01.tp.0000258649.23081.a3 17414713

[B38] Prescott R. S. Alsanea R. Fayad M. I. Johnson B. R. Wenckus C. S. Hao J. (2008). *In Vivo* generation of Dental Pulp-like Tissue by Using Dental Pulp Stem Cells, a Collagen Scaffold, and Dentin Matrix Protein 1 after Subcutaneous Transplantation in Mice. J. Endodontics 34, 421–426. 10.1016/j.joen.2008.02.005 PMC2408448 18358888

[B39] Rubio D. Garcia-Castro J. Martín M. C. de la Fuente R. Cigudosa J. C. Lloyd A. C. (2005). Spontaneous Human Adult Stem Cell Transformation. Cancer Res. 65, 3035–3039. 10.1158/0008-5472.can-04-4194 15833829

[B40] Seo B.-M. Miura M. Gronthos S. Mark Bartold P. Batouli S. Brahim J. (2004). Investigation of Multipotent Postnatal Stem Cells from Human Periodontal Ligament. The Lancet 364, 149–155. 10.1016/s0140-6736(04)16627-0 15246727

[B41] Seo B. Sonoyama W. Yamaza T. Coppe C. Kikuiri T. Akiyama K. (2008). SHED Repair Critical-Size Calvarial Defects in Mice. Oral Dis. 14, 428–434. 10.1111/j.1601-0825.2007.01396.x 18938268 PMC2653202

[B42] Sonoyama W. Liu Y. Fang D. Yamaza T. Seo B.-M. Zhang C. (2006). Mesenchymal Stem Cell-Mediated Functional Tooth Regeneration in Swine. PLoS One 1, e79. 10.1371/journal.pone.0000079 17183711 PMC1762318

[B43] Sonoyama W. Liu Y. Yamaza T. Tuan R. S. Wang S. Shi S. (2008). Characterization of the Apical Papilla and its Residing Stem Cells from Human Immature Permanent Teeth: a Pilot Study. J. Endodontics 34, 166–171. 10.1016/j.joen.2007.11.021 PMC2714367 18215674

[B44] Stevens A. Zuliani T. Olejnik C. LeRoy H. Obriot H. Kerr-Conte J. (2008). Human Dental Pulp Stem Cells Differentiate into Neural Crest-Derived Melanocytes and Have Label-Retaining and Sphere-Forming Abilities. Stem Cell Dev. 17, 1175–1184. 10.1089/scd.2008.0012 18393638

[B45] Taba M. Jr. Jin Q. Sugai J. Giannobile W. (2005). Current Concepts in Periodontal Bioengineering. Orthod. Craniofac. Res. 8, 292–302. 10.1111/j.1601-6343.2005.00352.x 16238610 PMC2581520

[B46] Takahashi K. Okita K. Nakagawa M. Yamanaka S. (2007). Induction of Pluripotent Stem Cells from Fibroblast Cultures. Nat. Protoc. 2, 3081–3089. 10.1038/nprot.2007.418 18079707

[B47] Takahashi K. Tanabe K. Ohnuki M. Narita M. Ichisaka T. Tomoda K. (2007). Induction of Pluripotent Stem Cells from Adult Human Fibroblasts by Defined Factors. Cell 131, 861–872. 10.1016/j.cell.2007.11.019 18035408

[B48] Trubiani O. Orsini G. Zini N. Di Iorio D. Piccirilli M. Piattelli A. (2008). Regenerative Potential of Human Periodontal Ligament Derived Stem Cells on Three-Dimensional Biomaterials: a Morphological Report. J. Biomed. Mater. Res. 87A, 986–993. 10.1002/jbm.a.31837 18257082

[B49] Vêncio E. F. Pascal L. E. Page L. S. Denyer G. Wang A. J. Ruohola-Baker H. (2011). Embryonal Carcinoma Cell Induction of miRNA and mRNA Changes in Co-cultured Prostate Stromal Fibromuscular Cells. J. Cel. Physiol. 226, 1479–1488. 10.1002/jcp.22464 PMC3968429 20945389

[B50] Waddington R. J. Youde S. J. Lee C. P. Sloan A. J. (2009). Isolation of Distinct Progenitor Stem Cell Populations from Dental Pulp. Cells Tissues Organs 189, 268–274. 10.1159/000151447 18701814

[B51] Warren L. Manos P. D. Ahfeldt T. Loh Y.-H. Li H. Lau F. (2010). Highly Efficient Reprogramming to Pluripotency and Directed Differentiation of Human Cells with Synthetic Modified mRNA. Cell Stem Cell 7, 618–630. 10.1016/j.stem.2010.08.012 20888316 PMC3656821

[B52] Wolf D. L. Lamster I. B. (2011). Contemporary Concepts in the Diagnosis of Periodontal Disease. Dental Clin. North America 55, 47–61. 10.1016/j.cden.2010.08.009 21094718

[B53] Yan X. Qin H. Qu C. Tuan R. S. Shi S. Huang G. T.-J. (2010). iPS Cells Reprogrammed from Human Mesenchymal-like Stem/progenitor Cells of Dental Tissue Origin. Stem Cell Dev. 19, 469–480. 10.1089/scd.2009.0314 PMC2851830 19795982

[B54] Yu J. Vodyanik M. A. Smuga-Otto K. Antosiewicz-Bourget J. Frane J. L. Tian S. (2007). Induced Pluripotent Stem Cell Lines Derived from Human Somatic Cells. Science 318, 1917–1920. 10.1126/science.1151526 18029452

[B55] Zaman K. U. Sugaya T. Kato H. (1999). Effect of Recombinant Human Platelet-Derived Growth Factor-BB and Bone Morphogenetic Protein-2 Application to Demineralized Dentin on Early Periodontal Ligament Cell Response. J. Periodontal Res. 34, 244–250. 10.1111/j.1600-0765.1999.tb02250.x 10567947

